# Music Recognition Using Blockchain Technology and Deep Learning

**DOI:** 10.1155/2022/7025338

**Published:** 2022-08-08

**Authors:** Xize Chen, Xiaoyu Qu, Yufeng Qian, Yiyao Zhang

**Affiliations:** ^1^School of Music and Dance, Qiqihar University, Qiqihar 161000, China; ^2^School of Science, Hubei University of Technology, Wuhan 430068, China; ^3^School of Art and Communication, Beijing Normal University, Beijing 100875, China

## Abstract

The purposes are to recognize and classify different music characteristics and strengthen the copyright protection system for original digital music in the big data era. Deep learning (DL) and blockchain technology are applied and researched herein. Based on CNN (Convolutional Neural Network), a music recognition method combined with hashing learning is proposed. The error generated when outputting the binary hash code is considered, and the semantic similarity of the hash code is ensured. Besides, the application of blockchain technology in the current intellectual property protection in original music is discussed. According to digital music property rights protection needs, the system is divided into modules, and its functions are designed. The system ensures its various functions by applying the application protocol designed in the Algor and network. In the experiments, the MagnaTagATune dataset is selected to verify the performance of the proposed CRNNH (Convolutional Recurrent Neural Network Hashing) algorithm. The algorithm shows the best music recognition performance under different bit numbers. When the number of connections is about 100, the QPS value of the blockchain-based music property rights protection system can be stabilized at about 20,000. At any number of threads, the system pressure will increase dramatically with the increase in the number of analog connections. The music recognition algorithm based on DL and hash method discussed is of great significance in improving the classification accuracy of music recognition. The application of blockchain technology in the copyright protection platform of original music works can protect the copyright of digital music and ensure the operation performance of the system.

## 1. Introduction

With the swift progress of the Internet, digital music can be widely distributed through different media. It refers to musical works stored in digital form, which can be created, edited, and played through music editing software. It is more flexible and convenient than traditional records [1]. It includes radio broadcasts and digital storage devices. People gradually have the opportunity to get in touch with different music from all over the world in the form of digital music, bringing a richer music experience to listeners of different music styles [2, 3]. The storage methods of music files in digital music have gradually become diversified. The optimization of music storage methods and the development of computer technology have also greatly improved digital audio processing technology [4]. The way of processing music is also gradually diversified, such as the sound recorder that comes with the Windows system and some libraries included in the Python language for processing music. As far as the current digital music processing technology is concerned, technologies such as speech recognition, text conversion, and speech compression coding are gradually produced with computer technology [5, 6]. After digital audio-based vectorization, musical features are stored and transmitted in digital form. Music in various formats can be displayed in different digital forms, making music analysis and processing accurate and efficient.

Since deep learning (DL) is a hot emerging technology for feature extraction today, improvements have been made to traditional deep neural networks. A music recognition method combined with hash learning is proposed. DL-based music recognition has practical significance for detecting and protecting original music. On this basis, the application of blockchain in the current intellectual property protection of original music is discussed to deepen the protection of digital music copyright. The distributed storage of musical compositions is analyzed so that the overhead of extracting data from the blockchain network to the storage network is as small as possible. In the end, the music property rights protection system by the blockchain algorithm is tested, which proves the usability of the system. The results manifest that the blockchain saves music recognition algorithm by the DL and the established neural network model. Many music users are only interested in certain styles of music, and music style recognition just classifies music into different types according to styles, allowing them to use the music recommendation function in line with their interests. It is convenient for users to quickly search and efficiently manage their favorite music. From this, the current work aims to identify and classify different music characteristics, strengthen the copyright protection of original digital music in the era of big data, and explore the application of DL and blockchain technology in this regard.

## 2. Related Works

To effectively retrieve and manage the music that end-users are interested in, music information retrieval (MIR) technology emerges as the times require. As a developing research field in multimedia systems, MIR has received great attention from the music industry [7]. The premise of music style recognition is a series of music style recognition and generation. The most influential techniques cover feature extraction and various classifiers. Different musical feature vectors used to identify musical styles will lead to different classification effects. Currently, the most common and reliable feature vectors are timbre, pitch, and loudness [8]. By adding music features, the researchers improved the recognition effect of style from the perspective of the signal generation principle, thereby improving the accuracy of music classification. However, there is still room for improvement in music recognition and classification results if only artificial feature extraction is employed. Deeper mining of the associations between the data is necessary for more accurate identification effects. DL has achieved great success in many fields and has also been widely accepted in the field of MIR. DL models are widely adopted in music generation. Solanki and Pandey [9] completed a deep Convolutional Neural Network (CNN) framework for identifying the main instruments in actual polyphonic music. The recognition accuracy of the instrument can reach 92.8% [9]. As an influential part of DL, Recurrent Neural Network (RNN) has made great breakthroughs in processing long-term sequences in music generation. The position of a note in the staff is one of the keys to pitch recognition. Andrea and Paoline [10] proposed a DL and CNN approach to identify note positions in musical notation [10]. By understanding the pitch of a note, taking the note image as input and the note position as output, pitch recognition can be achieved effectively and accurately. Alfaro-Contreras and Valero-Mas [11] considered the application of two convolutional RNN schemes trained to extract information from musical scores [11]. Aiming at the problem of identifying the shape of music symbols and their vertical positions in staves, an end-to-end identification method is proposed.

Two or three stages of convolution, nonlinear transformation, and pooling are concatenated, followed by more convolution and fully connected layers. The backpropagation algorithm of CNN is the same as that of a general deep network, which can train the ownership values of all filters. The key of CNN training with a genetic algorithm is how to map the network to chromosomes. In traditional coding mode, each network parameter is regarded as an element of the chromosome. In a large-scale network, this coding mode will make the chromosome structure too large and lead to the failure of genetic operators. One solution is to treat each convolution layer and the fully connected layer as a whole as an element of the chromosome. That is, each chromosome element contains either all the connection weights of a layer or all the values of a filter.

There are loads of original music content and works in the classification and promotion of digital music recognition. The rise of digital content operations has accelerated the diversified development of the content industry. However, at the same time, problems with the management and protection of digital copyright have become undeniable, and digital copyright infringements have increasingly become the sharp focus of the Internet industry [12]. Blockchain technology applies to a series of applications such as content creation, source authentication, and copyright protection, involving many industries such as news media, digital media, film and television, and games [13]. Blockchain technology has many techniques and branches, divided into public chains, alliance chains, and private chains according to access and management permissions. In the field of intellectual property protection, a technology that can be transparent and open and has high credibility is needed to promote intellectual property protection [14, 15]. The stability of content communication depends on the coding method under the insertion of Lagrangian polynomials. When enough coding items are obtained, new coding items can be generated and shared.

## 3. Method

### 3.1. Music Style Recognition Based on Deep CNN

**Figure 1:**
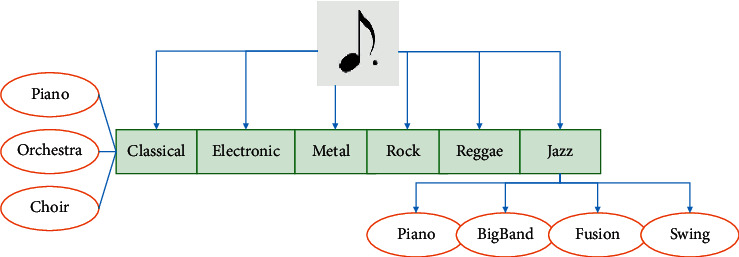
Ismir2004 Genre music classification structure.

Music can be divided into multiple genres according to different musical characteristics. Music genre refers to the unique musical style formed under the long-term mutual influence of the market and artists. Ismir2004 Genre structure can classify music genres. The structure divides music genres into 6 categories: Classical, Electronic, Reggae, Jazz/Blues, Metal/Punk, and Rock/Pop. Each of the different genres contains its representative instruments. Ismir2004 genre music classification structure is illustrated in Figure 1.

Extracting music features is particularly important to distinguish different music genres and styles [16]. Musical characteristics can reveal the essential attributes of music, and the most basic division of musical characteristics is based on human auditory experience. Musical characteristics can be divided into three subjective attributes: timbre, loudness, and pitch. Timbre is a characteristic attribute that can distinguish two identical tones; for example, different musical instruments have different timbre and tone quality. Loudness can show the intensity of playing a note. Pitch can exhibit the frequency of the sound. Another type of music feature classification is to divide music features into short-term features and long-term features. The above three subjective attributes can all be represented by precise numerical features; thus, they are short-term features. What cannot be expressed with numerical values is the time-domain characteristics. For example, short-term energy can express the amplitude of a music signal at a particular moment. Its equation is as follows:

(1) $$\begin{array}{c} E_{n}=\sum_{m=-\infty }^{\infty }{\left[x\left(m\right)w\left(n-m\right)\right]}^{2}\\ =\sum_{m=n-\left(N-1\right)}^{n}{\left[x\left(m\right)w\left(n-m\right)\right]}^{2}. \end{array}$$

In (1), *n* refers to the *n*-th sampling point, *N* stands for the window length, and *w*(*n* − *m*) denotes the window function.

The short-time average zero-crossing rate is a characteristic parameter in the time-domain analysis of speech signals. It refers to the number of times the signal passes the zero value in each frame. The zero-crossing rate can indicate the frequency information of the signal. When analyzing the waveform, the more high-frequency components, the more times the zero-point is crossed. The equation for this characteristic parameter is as follows:

(2) $$\begin{array}{c} Z_{n}=\frac{1}{2N}\sum_{m=n-\left(N-1\right)}^{n}\mathrm{sgn}\left[x\left(m\right)-\mathrm{sgn}x\left(m-1\right)\right]w\left(n-m\right), \end{array}$$

(3) $$\begin{array}{c} \text{sgn}\left[x\left(n\right)\right]=\left\{\begin{array}{cc} 1, & x\left(n\right)\geq 0,\\ -1, & x\left(n\right)<0. \end{array}\right. \end{array}$$

In (2) and (3), *x*(*m*) refers to the signal value of the *m*-th sampling point, and sgn stands for the sign function.

The short-time average zero-crossing rate can judge voice signals. If the zero-crossing rate is high, the voice signal will be unvoiced; if the zero-crossing rate is low, the voice signal will be voiced. Besides, it can also analyze music characteristics with frequency-domain features, including spectral centroid and spectral energy. The equation of spectrum energy can be expressed as follows:

(4) $$\begin{array}{c} \text{SE}=\sqrt{\frac{1}{\left(h_{0}-l_{0}\right)}}\sum_{w-l_{0}}^{h_{0}}{\left|F\left(W\right)\right|}^{2}. \end{array}$$

In (4), *l*_0_ refers to the minimal value of frequency, *h*_0_ refers to the maximum value of frequency, and *h*_0_ > *W* > *l*_0_.

The spectrum centroid is the gravity center of the frequency components, the energy-weighted average frequency within a given frequency range. Its equation can be expressed as follows:

(5) $$\begin{array}{c} \text{SC}=\frac{\sum_{w=l_{0}}^{h_{0}}w{\left|F\left(W\right)\right|}^{2}}{\sum_{w=l_{0}}^{h_{0}}{\left|F\left(W\right)\right|}^{2}}. \end{array}$$

According to the theoretical basis of the above musical characteristics, the recognition effect of the musical style can be improved by enhancing the musical characteristics. However, in some special cases, it is challenging to accurately extract music features manually, making it necessary to deeply mine into the internal connections of data. Machine learning technology can discover the structures of images or audios and express them as features through algorithms, which effectively overcomes the limitations of manually extracting features. In particular, hierarchical representation learning based on CNN has gradually been accepted in music style classification and music attribute recognition [17]. If only relying on manual feature extraction, the results and efficiency of classification will be disappointing. To get more accurate recognition results, it is necessary to deeply mine the internal association between data. As a preprocessing step in machine learning, feature extraction is very effective in reducing the dimension, removing irrelevant data, increasing learning accuracy, and improving the comprehensibility of results. The essence of feature extraction is clustering. In order to find a fast feature selection method, the effect must be effectively identifying the data irrelevance and redundancy, and the computational complexity should be low. In this sense, feature extraction is based on finding the appropriate correlation measure between features and the feasible feature selection steps based on this measure.

**Figure 2:**
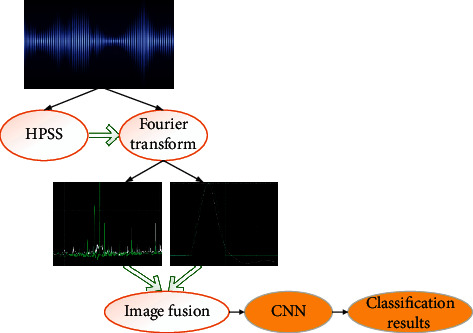
CNN-based music style recognition process.

The CNN architecture is very similar to the conventional ANN (Artificial Neural Network) architecture, especially the last layer of the network: the fully connected layer. CNN can accept multiple feature maps as input instead of vectors. The workflow of CNN is as follows. An image is sent to the model and goes through some convolutional, nonlinearization (activation function), pooling, and fully connected layers; finally, the result is obtained [18]. First, the music is separated by the HPSS (harmonic/percussion) algorithm. The original music is separated into the harmonic sound sources and the percussion sound sources, which undergo the STFT (Short-Time Fourier Transform). Subsequently, the transformed spectrogram is input into CNN for learning, and the output result is the final music feature recognition rate. The overall framework of CNN-based music style recognition is displayed in Figure 2.

The HPSS algorithm is a spectrogram-based signal separation. Furthermore, the separation is based on the continuous directional difference between the harmonic spectrum and the percussion spectrum [19]. The separated harmonic spectrum is a continuous and smooth distribution along the time axis at a fixed frequency; in contrast, the separated percussion spectrum is a continuous and smooth distribution along the frequency axis on the time axis. The original spectrum *W*_*f*,*t*_ is split into a percussion spectrum *P*_*f*,*t*_ and a harmonic spectrum *H*_*f*,*t*_:

(6) $$\begin{array}{c} W_{f,t}=\left(P_{f,t}+H_{f,t}\right). \end{array}$$

In (6), *f* and *t* represent the frequency index and time index, respectively, and *P*_*f*,*t*_ and *H*_*f*,*t*_ refer to the STFT of percussion and harmony, respectively. Minimizing (6) separates percussion and harmonics.

(7) $$\begin{array}{c} Q\left(H^{i},P^{i},U^{i},V^{i}\right)=\frac{1}{\sigma_{H}^{2}}\sum_{f,t}\left\{{\left(H_{f,t-1}^{i}-U_{f,t}^{i}\right)}^{2}-{\left(H_{f,t}^{i}-U_{f,t}^{i}\right)}^{2}\right\} \end{array}$$

(8) $$\begin{array}{c} V_{f,t}^{i+1}=\frac{1}{2}\left(P_{f-1,t}^{i}+P_{f,t}^{i}\right), \end{array}$$

(9) $$\begin{array}{c} U_{f,t}^{i+1}=\frac{1}{2}\left(H_{f-1,t}^{i}+H_{f,t}^{i}\right). \end{array}$$

In (7)–(9), *I* expresses the current iteration number and is an auxiliary parameter. 1/*σ*_*H*_^2^ and 1/*σ*_*P*_^2^ represent the smoothness parameters of harmony and percussion, respectively. The variables in (7) need to be updated to obtain the minimum value. The update equations are displayed in (10) and (11):

(10) $$\begin{array}{c} H_{f,t}^{i+1}=H_{f,t}^{i}+\Delta^{i}, \end{array}$$

(11) $$\begin{array}{c} P_{f,t}^{i+1}=P_{f,t}^{i}+\Delta^{i}, \end{array}$$

(12) $$\begin{array}{c} \Delta^{i}=\frac{\alpha }{4}\left(H_{f,t-1}^{i}+2H_{f,t}^{i}+H_{f,t+1}^{i}\right) \end{array}$$

In (10)–(12), Δ^*i*^ is the auxiliary parameter, and *α* is the weighting factor. After many iterations, the target equation approaches the minimum, and finally, the music signal is separated into harmonic sound and percussion sound.

**Figure 3:**
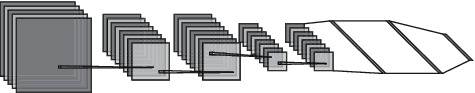
A schematic diagram of CNN structure for music style recognition.

The harmonic spectrum, percussion spectrum, and original music signal spectrum obtained after HPSS separation are input into the CNN network as input images. The image features are automatically extracted through the first few layers of CNN. The softmax function is used for classification and recognition in the last layer. In the deep network structure, the first convolutional layer uses 96 convolution kernels with a size of 11 × 11 to filter the input image. The second convolutional layer is connected to the first convolutional layer's output and uses 256 5 × 5 convolution kernels for filtering. The third, fourth, and fifth convolutional layers are connected to each other. Finally, 256 6 × 6 feature maps are obtained and fed to three fully connected layers. The output of the last fully connected layer is the final music style recognition result Figure 3.

The convolutional layer generates a feature map through a linear convolution filter and a nonlinear activation function. The output of neurons in the same layer forms a plane; that is, the feature map. Then, the convolutional feature maps are obtained through pooling and filtered to the next layer. Different kernel filters are set in the receptive field to obtain different feature maps.

### 3.2. Music Recognition Combining Hashing Learning

At present, most deep hashing learning methods are based on manually extracted features, making them unsuitable for hash coding learning [20–22]. Therefore, a deep hash method based on feature learning is proposed, which combines feature learning with hash coding learning to obtain higher accuracy.

**Figure 4:**
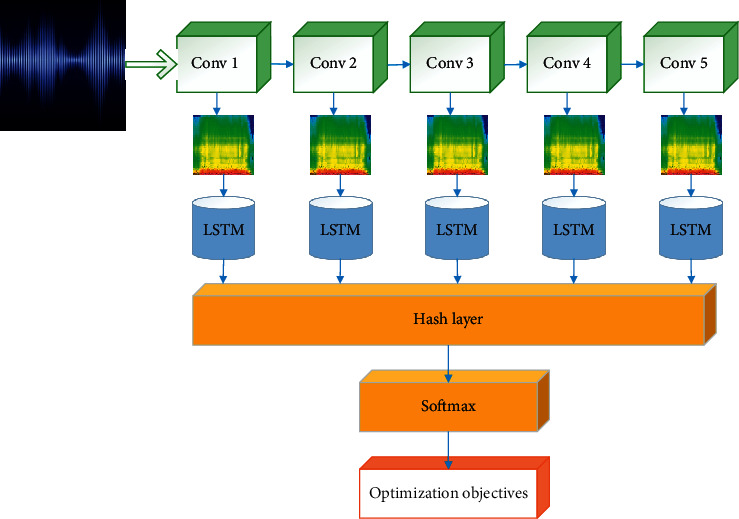
DL structure of music recognition combining hashing learning.

The proposed DL structure, combined with hashing learning, for music recognition is presented in Figure 4. First, the music signals are preprocessed, including STFT and logarithm of the spectrogram amplitude. The preprocessed image is input into a pretrained 5-layer CNN, and the convolutional feature map is extracted. The feature map sequence of each convolutional layer is obtained through bilinear interpolation and similarity selection. The sequence is then input into the LSTM (Long Short-Term Memory) network and the hash layer for recognition and classification.

LSTM is an improvement of RNN, which uses new units to replace the nonlinear units of traditional RNNs. LSTM includes a memory unit and three gates (input gate, forget gate, and output gate) [23]. The memory unit saves the input records. The input gate controls whether the LSTM reads the current input. The forget gate determines how much of the unit state from the last moment is retained to the current moment. The output gate controls how much of the current unit state is output as the current output value in LSTM. If the input sequence is *x*={*x*_1_, *x*_2_,…, *x*_*i*_}, the memory unit *c*, input gate *i*, forget gate *f*, and output gate *o* are expressed as follows:

(13) $$\begin{array}{c} c_{t}=f_{t}⊙c_{t-1}+i_{t}⊙\phi \left(W_{xc}x_{t}+W_{hc}x_{t-1}+\upsilon_{c}\right), \end{array}$$

(14) $$\begin{array}{c} i_{t}=\tau \left(W_{xi}x_{t}+W_{hi}h_{t-1}+W_{hi}c_{t-1}+\upsilon_{i}\right), \end{array}$$

(15) $$\begin{array}{c} f_{t}=\tau \left(W_{xf}x_{t}+W_{hf}h_{t-1}+W_{cf}c_{t-1}+\upsilon_{f}\right), \end{array}$$

(16) $$\begin{array}{c} o_{t}=\tau \left(W_{xo}x_{t}+W_{ho}h_{t-1}+W_{co}c_{t-1}+\upsilon_{o}\right), \end{array}$$

(17) $$\begin{array}{c} h_{t}=o_{t}⊙\phi \left(c_{t}\right). \end{array}$$

In (13)–(17), *t* refers to the time step, *τ*( ) denotes the sigmoid function, *ϕ*( ) represents the tanh function, *υ*_*∗*_ stands for the bias, *h*_*t*_ indicates the hidden layer output value, *W*_*x*^*∗*^_ refers to the weight between the input and the LSTM unit, *W*_*h*^*∗*^_ refers to the weight between the hidden states, and *W*_*c*^*∗*^_ refers to the weight between the memory unit and the gate.

After the convolutional feature map sequence is input into the first LSTM, a feature vector sequence *H*_abstrate_ can be obtained. The feature vector sequence is integrated through the second LSTM (LSTM__encode__^abstrate^), and the state of the last hidden layer of *LSTM*__encode__^abstrate^ can be expressed as follows:

(18) $$\begin{array}{c} h_{\text{end}}=LSTM_{{}_{\text{encode}}}^{\text{abstrate}}\left(H_{\text{abstrate}},W_{2},\upsilon_{2}\right). \end{array}$$

In (18), *W*_2_ and *υ*_2_ represent the weight and bias of LSTM__encode__^abstrate^, respectively.

The hidden layer and hash layer of LSTM__encode__^abstrate^ are fully connected, followed by the tanh function. The hash code *q* can be defined as follows:

(19) $$\begin{array}{c} q=\phi \left(W_{H}^{T}h_{\text{end}}+\upsilon_{H}\right). \end{array}$$

In (19), *W*_*H*_ and *υ*_*H*_ represent the weight and bias of the hash layer, respectively.

CRNNH (Convolutional Recurrent Neural Network Hashing) can express the feature map sequence as a hash code. The threshold function is defined as follows in acquiring the binary code:

(20) $$\begin{array}{c} b=\text{sign}\left(q\right). \end{array}$$

In (20), sign ( ) represents the sign function of the element.

The generation of music sequence is mainly realized by a prediction algorithm. In the process of prediction, according to the input vector *I*[*X*_0_, *X*_1_, *X*_2_ … *X*_*n*_], the output vector *S* is obtained from the linear layer through forward propagation. The key code of the music sequence generation algorithm is shown in Algorithm 1. Only some genres of music generation code (jazz and classical music) are shown in the code, and other genres have the same method of generating music sequence.

### 3.3. Blockchain Platform Algorand

**Figure 5:**
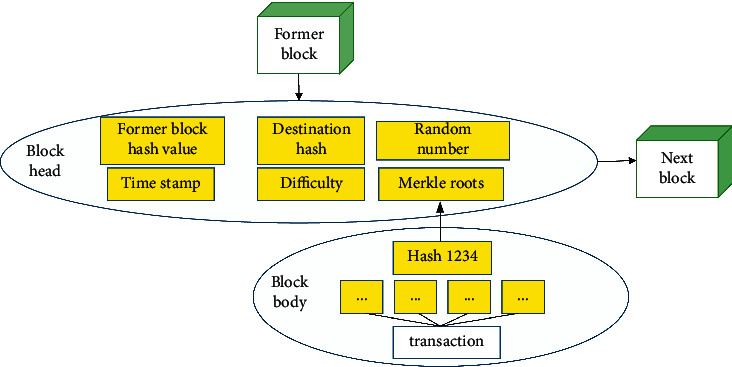
Block structure.

The blockchain is actually a noncentral distributed database, which records all transaction records from the operation of the blockchain network to the present and can be viewed in an authorized manner [24]. The block structure includes two parts: block head and block body, as shown in Figure 5. The blockhead contains the information that constitutes the blockchain, such as the hash value, timestamp, and the root of the Merkle tree of the block bodies. The block body stores multiple transaction information packaged into the block.

**Figure 6:**
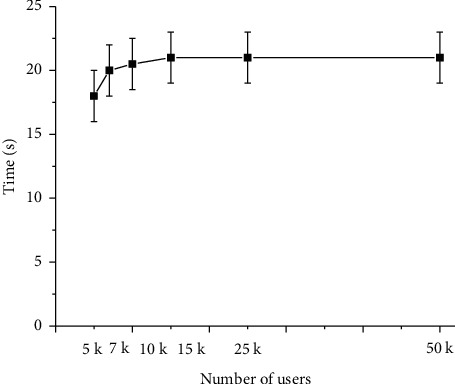
Algorand confirmed transaction time under different number of users.

Algorand is a blockchain protocol proposed by Professor Silvio Micali in 2016 to solve the problem of blockchain “Impossible Triangle.” The “Impossible Triangle” means that the blockchain cannot guarantee scalability, decentralization, and security at the same time. Algorand is a compound word of algorithm and random, essentially a public ledger protocol based on random algorithms [25]. Algorand is a public chain system. Users can join Algorand at any time without prior application. Algorand also has no restrictions on the number of users, indicating that each user holds multiple public keys, and each public key has a corresponding private key. Password lottery is a key innovation of Algorand. Algorand creates and continuously updates an independent parameter called “seed” [26]. The “seed” parameter cannot be predicted or manipulated by the “adversaries.” MIT (Massachusetts Institute of Technology) Computer Science and Artificial Intelligence Laboratory tested the Algorand through simulations. Algorand can confirm transactions within 1 minute, and the time consumption of transaction confirmation increases with the number of users. The results are displayed in Figure 6 [27].

### 3.4. Digital Music Property Rights Protection System Based on Blockchain

**Figure 7:**
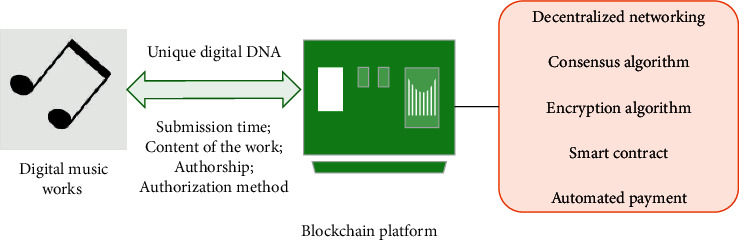
Music copyright determination system based on blockchain technology.

The life cycle of digital music products includes creation, dissemination, and consumption links [28]. The blockchain technology can store the personal information, time information, and music content of the copyright owner of the work in each block, and the unique authentication DNA of the music work can be generated through cryptography. In this process, third-party intermediaries are no longer needed [29, 30]. The detailed copyright determination system can be summarized as the process in Figure 7. The blockchain can directly provide nontamperable certificate tracking records, thereby ensuring the security of the copyright determination stage.

**Figure 8:**
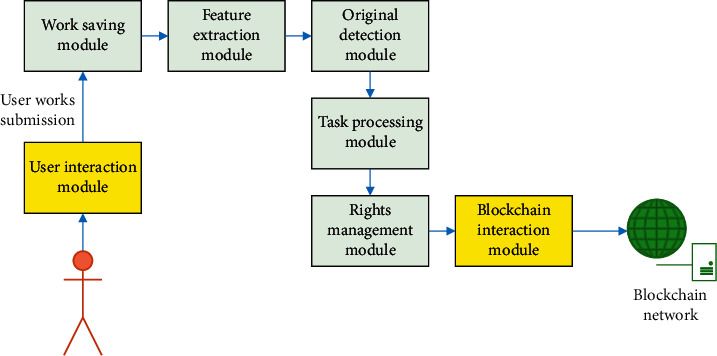
Organizational process among the modules of the system.

According to digital music property rights protection needs, the system is divided into several modules, and its functions are designed. First, the digital music property rights protection system is built on the public blockchain network to ensure the disclosure of music content and the effective protection of copyright information. Second, the CRNNH feature extraction algorithm proposed above can resist malicious behaviors, such as repetition and plagiarism, in the original detection link, thereby protecting the original music works in a true sense. In addition, this system ensures its various functions by applying the application protocol designed in the Algorand network. The music property rights protection system consists of four modules: local database, front-end, back-end, and blockchain. The basic process of system operation is as follows. Users log in after registration, and the system saves the account, password, and other data in the local database. Users can uplink their works on the chain. The music is compressed by feature extraction algorithms and converted into strings for the hash operation to obtain a hash value. Finally, this hash value is passed to the blockchain, and the original work is stored in a distributed database [31, 32]. The function of the music property rights protection system based on the blockchain platform consists of 7 modules: user interaction module, feature extraction module, blockchain interaction module, original detection module, work preservation module, task processing module, and rights management module. The organizational process among the modules of the system is shown in Figure 8.

This system is essentially a Web application so that its implementation is separating the front and back ends of the B/S (Browser/Server). The front-end user interface is implemented using Ant Design of the front-end UI component library; the back-end server is developed using the Spring framework under the Java platform. The digital music property rights protection system designed on the Algorand public chain can provide effective protection for original authors and works.

### 3.5. Experiment and Parameter Setting

The MagnaTagATune dataset is selected for experiments to verify the music recognition effect of CNN combined with hash learning. The dataset contains 200 hours of music divided into segments every 28 seconds. Each clip is annotated with 188 tags (including musical genre, instrument, rhythm, volume, and mood). The dataset is highly imbalanced, with some labels having most of the data and much more content related to it than others. The dataset is split into 19773 training sets and 6087 testing sets. First, the dataset is preprocessed and the data is shuffled before creating the training and test sets. All MP3 songs are converted to their own Mel spectrum. It is very important for DL models to properly tune hyperparameters. It can be divided into two categories: model-related hyperparameters (Table 1) and training-related (Table 2).

The Hamming distance of the sample is calculated through the binary semantic features of music obtained by DL. The recognition rate of each test sample is calculated, and the final MAP (Mean Average Precision) is calculated. When the Hamming distance between the test sample and the training sample is less than 2, the percentage of correct results is calculated. The performance of the hash method can be comprehensively evaluated through the above two indicators.

The web pressure testing tool work is employed to initiate a test to an API interface in the background service to test the service performance of the protection system for blockchain-based digital music property rights. The system pressure is altered by changing the number of threads and the number of analog connections. Changes in various performance indicators of the property rights protection system are analyzed.

## 4. Results and Discussion

### 4.1. Influence of Different CNN Parameter Setting on Music Recognition

**Figure 9:**
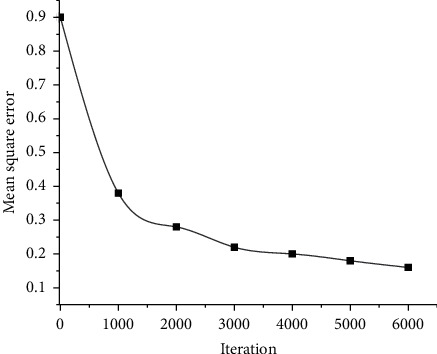
Neural network error with different numbers of hidden layer neurons.

When designing the neural network model, the influence of different LSTM iteration times on the music sequence generation is first analyzed to optimize the parameters of the model. The influence of the iteration times on the training effect is illustrated in Figure 9. As the number of iterations increases, the experimental error value gradually decreases. Especially, when the iteration increases from 0 to 2,000 times, the experimental error value decreases sharply. When the number of iterations reaches 6000, the error value at this point becomes flat. Considering that increasing the number of iterations will extend the training time, there is no need to increase the number of iterations.

**Figure 10:**
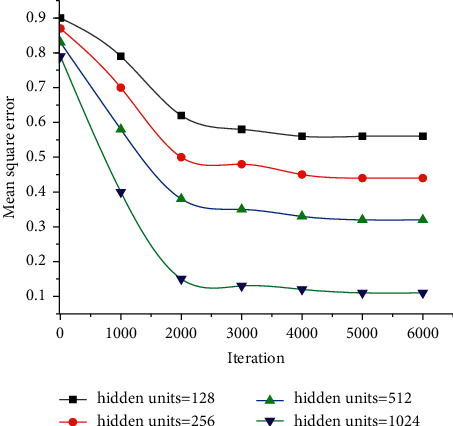
Neural network error with different numbers of hidden layer neurons.

On this basis, the influence of the number of hidden layer neurons on the model error is analyzed, and the results are shown in Figure 10. When the number of hidden layers is 4 (the number of neurons is 1024, 512, 256, and 128 layer by layer), the training result of the model has the highest accuracy, and the error value is the smallest. If the number of neurons increases continuously, higher computing performance is required, and the training time and complexity of the model will be considerably increased, which will reduce the training results of the network.

### 4.2. Music Recognition Effect Based on CRNNH Algorithm

**Figure 11:**
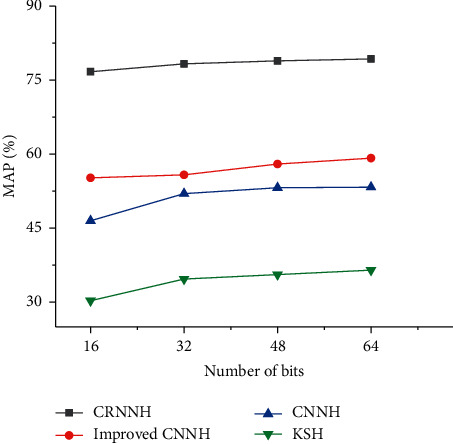
The influences of different hash bits on the results of music recognition.

**Figure 12:**
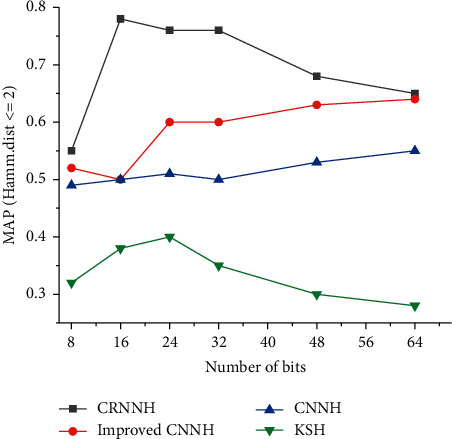
Accuracy curves with Hamming distance less than 2 under different bit numbers.

**Figure 13:**
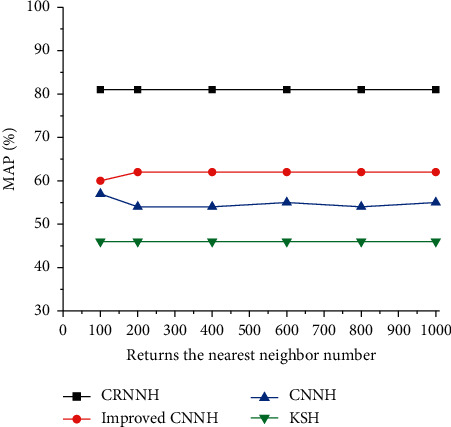
Retrieval results under 64-bit hash bits.

The influence of different hash bits in the CRNNH algorithm on the result of music recognition is shown in Figure 11. The traditional artificial feature learning method Kernel-based Supervised Hashing (KSH) is selected for comparative experiments. CNN features can improve the representation ability of music spectrograms by combining them with CNN in DL. Besides, the recognition accuracy is also significantly improved. Compared with other hashing methods, the CRNNH algorithm improves the MAP, which is related to its application of a new loss function to maintain the semantic similarity of hash codes. In addition, the CRNNH algorithm generates hash codes through convolutional feature maps containing spatial details and semantic information. Compared with several other supervised hashing methods, the recognition of images is more complete and comprehensive.  Figure 12 shows the accuracy curve of Hamming distance less than 2 when using different bit numbers. The CRNNH algorithm shows the best music recognition performance under different bit numbers. The accuracy under 64-bit hash bits is illustrated in Figure 13. Among all the algorithms, the recognition accuracy obtained by the CNNH algorithm is the best.

### 4.3. Performance Test of Music Property Rights Protection System Based on Blockchain

**Figure 14:**
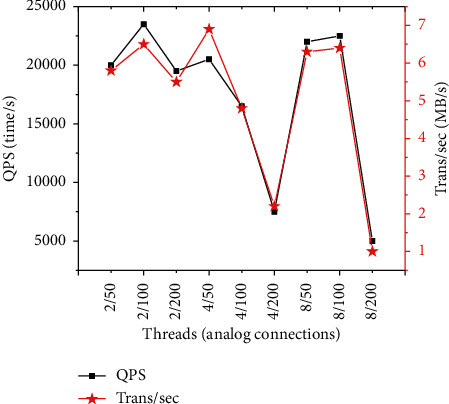
System performance test results under different threads/analog connections.

The music property rights protection system undergoes a pressure test, and the peak availability of services provided by the system during peak periods is recorded. The number of threads is set as 2, 4, and 8, and the number of analog connections is set to 50, 100, and 200, respectively, to test the QPS (Queries-per-second) and the data transmission rate (Trans/sec). The results are shown in Figure 14. Given any number of threads, the pressure on the system will increase dramatically with the increase in the number of analog connections. Due to the limitations of the current hardware conditions, the increase in the number of analog connections will significantly reduce the system QPS value. When the number of connections is about 100, the system QPS value can stabilize at about 20,000. This result means that the current peak performance of the server can meet the needs of 20,000 simultaneous users.

**Figure 15:**
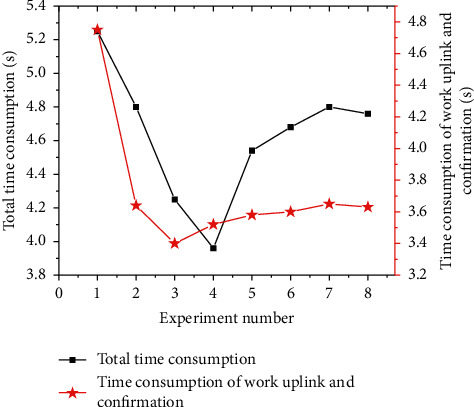
The total time consumption and the time consumption of work uplink and confirmation of the system.

Under the same operation process, eight sets of controlled trials are set up in different time periods. The operations are completed according to the same steps (user submits digital music works-music feature extraction and detection-works on the chain-confirmation of deposit). By adding the timing code to the background service, the total time consumption in the entire process is obtained, as well as the time consumption of work uplink and confirmation. The results are presented in Figure 15. At present, the total time consumption of the traditional Ethereum transaction process is about 5 minutes. In contrast, the total time consumption measured by eight experiments is basically about 4∼5 seconds, and the time consumption of the work uplink and confirmation process is controlled at about 4 seconds. The efficiency of the blockchain copyright protection system essentially lies in the excellent block generation speed and confirmation mechanism of the Algorand blockchain.

### 4.4. Experiment Results and Discussion

In summary, through the performance comparison of different CNN model parameters, the music recognition effect of the CRNNH algorithm is compared with the CNN model. The research results manifest that the current traditional Ethereum transaction process takes about 5 minutes in total. In contrast, the total time taken by the eight experiments is basically about 4–5 seconds, and the time-consuming of the work uplink and confirmation process is controlled at about 4 seconds. In addition, Li [33] conducted research on automatic piano composition and recognition technology based on DL and introduced the RNN, LSTM, and Gated Recurrent Unit (GRU) network and DL-based neural networks. The findings indicate that the GRU-RNN model shows satisfactory results in both manual analysis evaluation or paragraph pause analysis. Calvo-Zaragoza and Rizo [34] studied an end-to-end neuro-optical recognition technique for music recognition of musical scores. They investigated several considerations regarding the encoding of the output musical sequence, the convergence and scalability of the neural model, and the ability of this method to locate symbols in the input musical score. The results testify that the application of DL and blockchain technology in the field of music recognition can improve its accuracy. Pati et al. [35] analyzed advanced computing and intelligent engineering. By discussing new concepts, designs, and technological advances in this field, the goal of the research is to solve the dilemmas faced by cutting-edge hardware technologies and future communication technologies. It has vital reference value for the development of intelligent computing and music recognition. Therefore, the proposed music recognition model based on DL and blockchain can provide a theoretical reference for the rapid growth of intelligence in the field of music.

## 5. Conclusion

With the development of information technology and multimedia technology, a wide range of digital music can be obtained through different media. Hence, in-depth research on music information retrieval technology is necessary for effectively retrieving the music that users are interested in loads of music libraries. DL, as a classic technology for processing complicated and high-dimensional data in the big data context, has gradually been applied to nonimage speech fields.

Here, a music recognition method is proposed based on CNN, which uses multilayer RNN to generate hash codes. A new loss function ensures the semantic similarity of hash codes. The MagnaTagATune dataset is selected to verify the performance of the CRNNH algorithm, and results suggest that compared with other hash methods, CRNNH shows a better retrieval performance. The blockchain applications in the current intellectual property protection of original music are also discussed to protect digital music copyright effectively. A copyright protection system for original music works is designed based on the Algorand blockchain, and its operability is verified through tests. The high computational load of DL greatly affects the efficiency of hashing learning. Reducing the number of parameters to compress the hashing learning model is the next direction that deserves further attention.

## Figures and Tables

**Algorithm 1:**
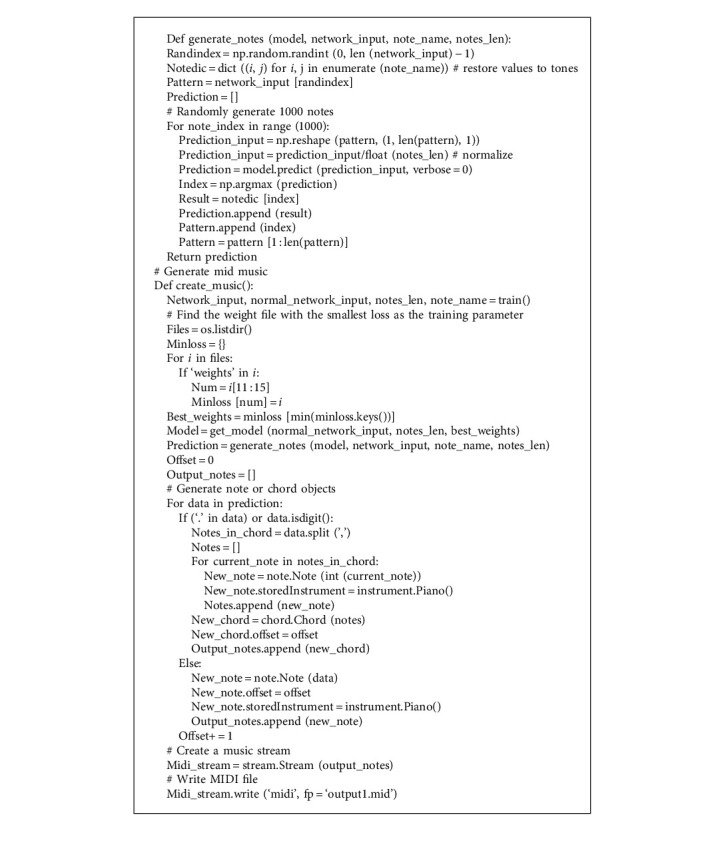
Key Code of Music Sequence Generation.

**Table 1:** Model-related hyperparameters.

Layers	Layer type	Kernel size	Pad	Size	Number of mapping layers
	Input			256^*∗*^256	1
1	Conv1	11^*∗*^11		55^*∗*^55	96
	Pool1	3^*∗*^3		27^*∗*^27	96

2	Conv2	5^*∗*^5	2	27^*∗*^27	256
	Pool2	3^*∗*^3		13^*∗*^13	256

3	Conv3	3^*∗*^3	1	13^*∗*^13	384
4	Conv4	3^*∗*^3	1	13^*∗*^13	384

5	Conv5	3^*∗*^3	1	13^*∗*^13	256
	Pool5	3^*∗*^3		6^*∗*^6	256

6	Fc6				4096
7	Fc7				1000
8	Fc8				10

**Table 2:** Training-related hyperparameters.

Hyperparameters	Learning rate	Batch-size	Momentum parameter	Weight attenuation coefficient	Dropout coefficient
Value	0.01	16	0.9	0.0005	0.5

## Data Availability

The raw data supporting the conclusions of this article will be made available by the authors without undue reservation.
